# Everything you ever wanted to know about cancer stem cells in neuroendocrine neoplasms but were afraid to ask

**DOI:** 10.1530/EO-24-0006

**Published:** 2024-12-19

**Authors:** Ignacio Ruz-Caracuel, Sergio Pedraza-Arevalo, Teresa Alonso-Gordoa, Javier Molina-Cerrillo, Julie Earl, Bruno Sainz

**Affiliations:** ^1^ Pathology Department, Hospital Universitario Ramón y Cajal, Madrid, Spain; ^2^ Molecular Pathology of Cancer Group, Area 3 Cancer, Instituto Ramón y Cajal de Investigación Sanitaria (IRYCIS), Madrid, Spain; ^3^ Centro de Investigación Biomédica en Red, CIBERONC, ISCIII, Madrid, Spain; ^4^ Maimonides Biomedical Research Institute of Córdoba (IMIBIC), Cordoba, Spain; ^5^ Department of Cell Biology, Physiology, and Immunology, University of Córdoba, Cordoba, Spain; ^6^ Reina Sofía University Hospital (HURS), Cordoba, Spain; ^7^ Medical Oncology Department, Hospital Universitario Ramón y Cajal, Madrid, Spain; ^8^ Biomarkers and Personalized Approach to Cancer (BIOPAC) Group, Area 3 Cancer, Instituto Ramón y Cajal de Investigación Sanitaria (IRYCIS), Madrid, Spain; ^9^ Department of Cancer, Instituto de Investigaciones Biomédicas (IIBm) Sols-Morreale (CSIC-UAM), Madrid, Spain

**Keywords:** cancer stem cell, neuroendocrine tumours, neuroendocrine neoplasia, neuroendocrine carcinomas

## Abstract

While the role of cancer stem cells (CSCs) in tumorigenesis, chemoresistance, metastasis, and relapse has been extensively studied in solid tumors, such as adenocarcinomas or sarcomas, the same cannot be said for neuroendocrine neoplasms (NENs). While lagging, CSCs have been described in numerous NENs, including gastrointestinal and pancreatic NENs (PanNENs), and they have been found to play critical roles in tumor initiation, progression, and treatment resistance. However, it seems that there is still skepticism regarding the role of CSCs in NENs, even in light of studies that support the CSC model in these tumors and the therapeutic benefits of targeting them. For example, in lung neuroendocrine carcinoids, a high percentage of CSCs have been found in atypical carcinoids, suggesting the presence of CSCs in these cancers. In PanNENs, CSCs marked by aldehyde dehydrogenases or CD90 have been identified, and targeting CSCs with inhibitors of molecular pathways has shown therapeutic potential. Overall, while evidence exists for the presence of CSCs in NENs, either the CSC field has neglected NENs or the NEN field has not fully embraced the CSC model. Both might apply and/or may be a consequence of the fact that NENs are a relatively rare and heterogeneous tumor entity, with confusing histology and nomenclature to match. Regardless, this review intends to summarize our current knowledge of CSCs in NENs and highlight the importance of understanding the role of CSCs in the biology of these rare tumors, with a special focus on developing targeted therapies to improve patients’ outcomes.

## Introduction: cancer stem cells

Today, the cancer stem cell (CSC) model is a well-accepted model to explain tumor heterogeneity, chemoresistance, metastasis, and tumor relapse following treatment cessation. Before the identification of CSCs, the clonal evolution (or stochastic) model was predominantly used for decades to explain tumor evolution and heterogeneity. This model suggested that tumors originated from a cell of origin, and acquisition of serial mutations in different tumor cells led to clonal evolution and tumor heterogeneity, in which all tumor cells could renew and had tumorigenic potential (Nowell 1976). This vision began to change between 1994 and 1997, starting with the seminal study by Dick and coworkers describing the first isolation and functional validation of CSCs from the bone marrow of patients with acute myeloid leukemia using the cell surface markers CD34 and CD38 via fluorescence-activated cell sorting (FACS) (Lapidot *et al.* 1994, Bonnet & Dick 1997). During the beginning of the 21st century, Al-Hajj and coworkers showed in 2003 that CD44+/CD24−/low breast cancer cells were CSCs (Al-Hajj *et al.* 2003), and in 2004, Dirks and coworkers showed that CD133+ cells from human brain tumors were tumor-initiating cells when injected into NOD-SCID mice (Singh *et al.* 2004). Between 2005 and 2007, CSCs were also identified in prostate and lung cancers by Collins and coworkers (Collins *et al.* 2005) and Kim and coworkers (Kim *et al.* 2005), respectively; in hepatocellular carcinoma by Suetsugu and coworkers (Suetsugu *et al.* 2006); in colon cancer by O’Brien and coworkers (O’Brien *et al.* 2007); in head and neck cancer by Prince and coworkers (Prince *et al.* 2007); and in pancreatic ductal adenocarcinoma by Li and coworkers (Li *et al.* 2007) and Hermann and coworkers (Hermann *et al*. 2007).

**Figure 1:**
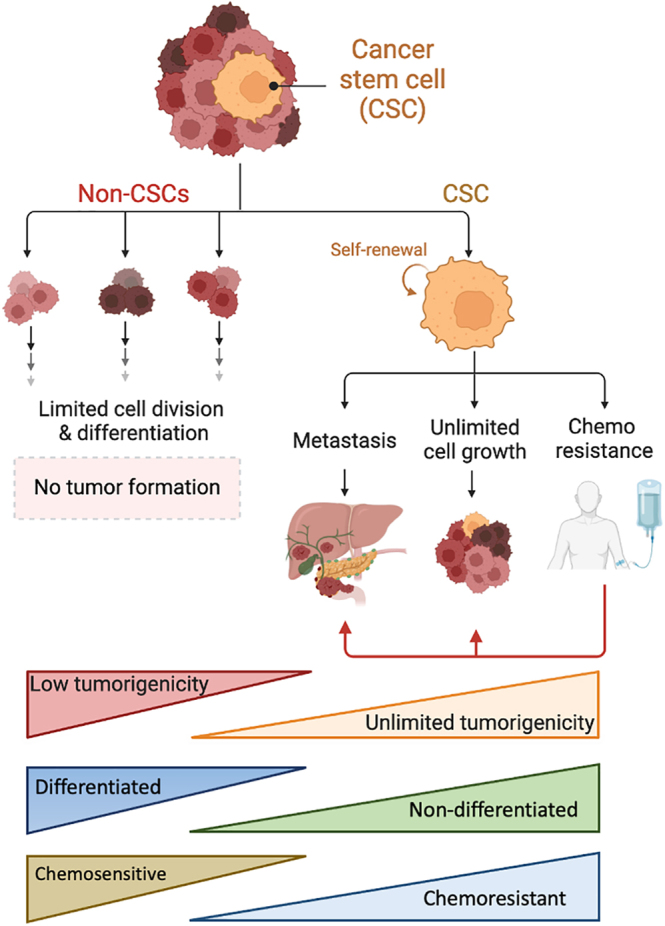
Cancer stem cell (CSC) model. CSCs represent a small subpopulation of the bulk cells present within the tumor. These cells can be separated or enriched based on physical (e.g., marker expression) or functional properties (e.g., sphere formation), respectively. In general, CSCs are different from their non-CSC counterparts at different levels. The main difference is that only CSCs have unlimited growth and tumorigenic potential. Similarly, CSCs are less differentiated than non-CSCs, have inherent metastatic capacity, and are highly chemoresistant such that following treatment cessation, if CSCs survive, they can drive tumor relapse. Figure created, in part, with BioRender.com.

Importantly, in 2006, a consensus panel made up of experts in the cancer field convened at the American Association of Cancer Research annual meeting to discuss CSCs in cancer, subsequently defining a CSC as ‘a cell within a tumor that possesses the capacity to self-renew and to cause the heterogeneous lineages of cancer cells that comprise the tumor’ (Clarke et *al.* 2006). Since then, and for the past 18 years, hundreds of published studies have provided evidence to support the 2006 CSC definition/model across different tumor types and entities, using both mouse and human systems (Fig. 1). The majority of these studies have one underlying approach in common to measure cancer stemness, the exclusive ability of a subpopulation of tumor cells to initiate tumors when transplanted *in vivo* (generally in immunocompromised mice).

For the aforementioned tumor initiation studies, researchers have primarily relied on FACS as the ‘gold-standard’ assay to isolate CSCs expressing a specific marker (or a combination thereof) followed by injection into immunocompromised mice to test the tumor-initiating capacity of the particular cell(s). Indeed, some reviews have questioned these approaches to isolate and functionally validate or characterize CSCs (Rahman *et al.* 2011, Lan & Behrens 2023), due in large part to the fact that CSCs represent a very small percentage of the bulk tumor cell population. The percentage of CSCs in a tumor varies depending on the tumor type but generally comprises a small population, for example, between 0.5 and 10%. Thus, their efficient isolation using flow cytometry-based techniques can be technically challenging. Similarly, since CSCs represent a small percentage of the bulk tumor cells, approaches to study these cells may sometimes be confounded by non-CSCs. Moreover, different CSC clones and their progenies may exist in a tumor at any given time, and no single CSC marker (as described below) will identify all CSC subpopulations. Thus, separation techniques may not always result in a pure non-CSC population, which needs to be taken into consideration in comparative analyses (i.e., CSC vs non-CSCs). In light of these obstacles, FACS and flow cytometry-based approaches using CSC-‘specific’/enriched biomarkers continue to be the most well-accepted techniques to isolate and/or identify CSCs in solid tumors, respectively, for downstream validation (e.g., tumorigenicity) and characterization (i.e., omic) studies.

Like their normal counterparts, CSCs in general have been shown to express certain cell surface and intracellular biomarkers, which include, but are not limited to, CD133 (PROM1), ABCG2, ALCAM (CD166), EphB2, ALDH1, CD44, CD29, CD24, CD90 (THY1), EpCAM (CD326), Integrin α6β4, c-KIT, c-MET, and LGR5 (Kapoor-Narula & Lenka 2022). Of these, the best studied CSC marker is undoubtedly the Wnt/β-catenin signaling molecule leucine-rich repeat-containing G-protein coupled receptor (LGR5), a marker of intestinal stem cells (ISCs) (Barker *et al.* 2007) and colon CSCs. Using colon cancer spheroid cultures of primary colorectal cancers and liver metastases, Vermeulen and coworkers were able to show in 2008 that the CD133+/CD24+ population were *bona fide* colorectal CSCs (Vermeulen *et al.* 2008), which also expressed LGR5. LGR5 as a CSC marker was confirmed in subsequent studies by Kanwar and coworkers, using colon spheres generated from established cell lines (Kanwar *et al.* 2010), and by Takahashi and coworkers, using patient-derived samples, to show that high LGR5 expression was associated with poor prognosis (Takahashi *et al.* 2011). Subsequently, Merlos-Suárez and coworkers, using murine colorectal tumor models, showed that the ISC program defines a CSC niche within colon cancer using the markers EphB2 and Lgr5 (Merlos-Suarez *et al.* 2011), and in lineage tracing experiments using human organoids, as shown by Cortina and coworkers (Cortina *et al.* 2017) and Oost and coworkers (Oost *et al.* 2018). Using LGR5-targeted antibody–drug conjugates, Gong and coworkers showed that targeting LGR5+ cells led to tumor eradication and prevention of disease recurrence in a xenograft model of colon cancer (Gong *et al.* 2016). Targeting LGR5+ CSCs was further demonstrated in genetically engineered mouse models by Sousa e Melo and coworkers (De Sousa E Melo *et al.* 2017) and Shimokawa and coworkers (Shimokawa *et al.* 2017). Thus, LGR5 represents the ‘ideal’ CSC marker for colon cancer as it can be used for CSC enrichment and lineage tracing, and targeting. However, while LGR5 has facilitated our understanding of CSCs in this tumor type over the past two decades, the role of LGR5+ CSCs in tumor relapse and metastasis has been challenged (Morral *et al.* 2020, Canellas-Socias *et al.* 2022), Therefore, CSC markers should be handled with caution, and a single marker may not identify CSCs that satisfy all CSC properties.

In addition to the aforementioned markers, CSCs are also defined by their ability to self-renew, produce differentiated progeny, and importantly, activate specific signaling, transcriptional, and/or epigenetic pathways and programs needed to maintain their stemness (Marquardt *et al.* 2018, Zeng *et al.* 2023). The evolving CSC model provides a valid explanation for the phenotypic and functional heterogeneity among cancer cells across many tumor types. It considers that tumors are hierarchically organized into subpopulations of tumorigenic ‘stem’-like cells (i.e., CSCs) and their non-tumorigenic progeny, with the CSC sitting at the apex, supporting and mediating cellular heterogeneity by establishing a differentiation hierarchy, which results in the various cancer cell types present within the tumor (Batlle & Clevers 2017, Hermann & Sainz 2018). For many years, this hierarchy was considered to be unidirectional, with the CSC representing a hard-wired entity, which, if eliminated, would result in tumor eradication. However, in 2017, two studies in colorectal cancer by de Sousa e Melo and coworkers (De Sousa E Melo *et al.* 2017) and Shimokawa and coworkers*.* Shimokawa *et al.* (2017) revealed that CSCs are not a hard-wired entity, but rather plastic/dynamic states, meaning that differentiated cells can also dedifferentiate and acquire CSC properties under specific conditions, mediated by signals often received from the CSC niche and/or tumor microenvironment (TME) (Lenos *et al.* 2018, Raghavan *et al.* 2021). Several studies since then have further validated this CSC bidirectional plasticity theory (McKenzie *et al.* 2019, Morral *et al.* 2020, Musella *et al.* 2022, Beziaud *et al.* 2023).

**Figure 2:**
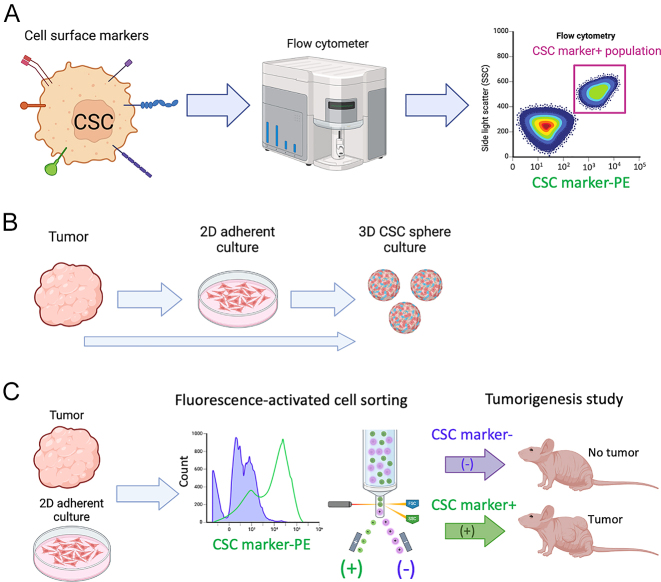
Methods to test for cancer stem cell (CSC) stemness *in vitro* and *in vivo*. (A) CSCs can be identified based on the expression of cell surface markers (for example). With the use of fluorescently labeled antibodies that can recognize these markers, CSCs can be identified from non-CSCs using techniques such as flow cytometry. (B) Adherent 2D cultures of tumor cells can be established from surgically resected tumors or patient-derived xenografts. These cultures contain a small percentage of CSCs. Culturing these cells under non-adherent conditions with specific media can favor the generation of spheres enriched in CSCs. Spheres can also be established directly from digested tumors. (C) The CSC gold standard assay is *in vivo* tumorigenesis. Using cell surface or intracellular markers, CSCs and non-CSCs can be sorted by fluorescence-activated cell sorting from digested tumors or adherent 2D cultures and the respective CSC marker-negative (−) and CSC marker-positive (+) populations can be injected into immunocompromised mice to assess their tumorigenic potential. Figure created, in part, with BioRender.com.

We can therefore conclude that CSCs i) are transformed stem-like cells with a series of genetic mutations and epigenetic modifications that allow them to activate pathways and programs (e.g., embryonic programs) necessary to maintain their stemness, self-renewal capacity, and chemoresistant nature, ii) undergo both symmetric and asymmetric division ensuring that the CSC pool is never lost and that tumor heterogeneity is maintained, and iii) have exclusive tumorigenic capacity. As previously mentioned, while many CSC markers have been identified and used to study, isolate, and identify CSCs (reviewed in Mohan *et al.* (2021) and Herreros-Pomares (2022)), a universal CSC marker is still lacking, and doubts regarding the specificity of CSC markers and their utility have been put forward (Lan & Behrens 2023). Nonetheless, the majority of approaches to study CSCs are based on surface marker expression using flow cytometry/FACS and, to a lesser extent, other separation methods, such as magnetically activated cell sorting, the ALDH-based ALDEFLUOR assay, or the Hoechst 33342 dye exclusion assay (i.e., side population). These separation approaches can be combined (or not) with *in vitro* and *in vivo* methods to better study the CSC population, including sphere formation, cell cycle, asymmetric division capacity, and drug resistance capacity (Ponomarev *et al.* 2022). Finally, tumorigenicity assays, including extreme limiting dilution assays, represent the gold standard to confirm that the cell(s) in question are *bona fide* CSCs. All of these features that define a CSC and the techniques used to characterize CSCs have recently been discussed in an excellent review by Loh and Ma, entitled ‘Hallmarks of cancer stemness’ (Loh & Ma 2024), and many are detailed in Fig. 2.

Without a doubt, the CSC concept has inspired the design of innovative treatment strategies to target these cells as a means of treating cancer. However, progress in the development of anti-CSC agents is very slow, and these types of compounds are still very far from reaching the clinic (reviewed in Chen *et al.* (2013), Ning *et al.* (2013), and Saygin *et al.* (2019)). Nonetheless, the knowledge gained from studying CSCs in diverse tumor entities has provided additional and useful information that has been used to better understand cancer in general. While true, it is clear that the study of CSCs in neuroendocrine neoplasms (NENs) has lagged behind hematological and other solid tumors (i.e., adenocarcinomas, sarcomas, etc.). For example, while CSCs were discovered in colon cancer by O’Brien and coworkers (O’Brien *et al.* 2007) in 2007, their discovery in gastrointestinal neuroendocrine tumors (NETs), the most common NEN, did not occur until 2011 (Gaur *et al.* 2011). It is unclear why CSCs in NENs have not been studied to the same degree as in other tumors, but factors such as the lack of *in vitro* models and the fact that NENs are a rare and highly heterogeneous tumor entity, with intricate histology and nomenclature to match, have likely influenced the study. Nonetheless, CSCs have been identified and studied in NENs. Masciale and coworkers used the ALDEFLUOR assay to identify ALDH high and ALDH low human lung cancer cells from a patient with an atypical carcinoid. The immunohistochemical analysis of SOX2 was also determined. The authors showed that more than half of the entire tumor cell population was composed of CSCs (Masciale *et al.* 2019).

Overall, while evidence exists for the presence of CSCs in NENs, the field has slowly advanced. A shift in this paradigm could potentially represent a significant step forward in understanding and possibly treating NENs. However, as detailed previously and throughout this review, it may be that we cannot readily apply the general rules, definitions, and hallmarks applicable for CSCs of other solids tumors to NENs, as these tumors are unequivocally very different and complex. Nonetheless, initially, it may be prudent to follow a similar path to try and understand these cells and ultimately determine how unique (and/or different) they are from other tumor cells and CSCs of other tumors. For this, we need to improve our understanding of CSCs in NENs at the following levels: i) discovery of precise and unique markers for NEN CSCs to differentiate them from other tumor cells and confirm the role of biomarker-positive NEN ‘CSCs’ at the functional level (e.g., self-renewal and tumor initiation); ii) understanding the role of CSCs in NEN evolution using gene editing approaches (e.g., CRISPR/Cas9-based genome editing techniques and lineage tracing); iii) unraveling the underlying molecular pathways that drive NEN CSC biology and behavior, including proliferation, survival, and metastasis using state-of-the-art omic-based techniques (e.g., single-cell RNA sequencing (scRNAseq) and/or spatial transcriptomics); iv) understanding the NEN CSC niche (i.e., TME) to investigate the role of nontumoral stroma cells in supporting CSC growth and function; v) developing targeted anti-CSC therapies that selectively target NEN CSCs, used alone or in combination with traditional therapies to simultaneously target both CSCs and bulk tumor cells; and vi) improving diagnostic tools based on the detection of NEN CSCs for early detection and/or monitoring disease progression.

By achieving advancements in these areas, through CSC-focused research, we will hopefully improve our overall understanding of NENs and develop more effective treatments for patients. Thus, in this review, we have summarized the current, but sparse, state of the knowledge of CSCs in NENs, highlighting what we know to date about the role of CSCs in the biology of these rare and complex tumors, with the hope of encouraging the community to continue to study CSCs in NENs.

## What are NENs?

NENs are a group of neoplasms originating from the neuroendocrine system, which is made up of specialized cells that secrete amine and peptide hormones into the bloodstream to target receptors nearby or in other parts of the body to regulate physiological processes (Asa *et al.* 2021). Neuroendocrine cells can form organs, such as the pituitary, the parathyroids, or the adrenals, or can be found as isolated cells or small islets in many organs, such as the thyroid, the lung, the gastrointestinal tract, or the pancreas, in what is called a diffuse neuroendocrine system. Due to its function, the neuroendocrine system was also called the ‘APUD system’, terminology used by Pearse (Pearse 1977) to refer to amine precursor uptake and decarboxylation.

**Figure 3:**
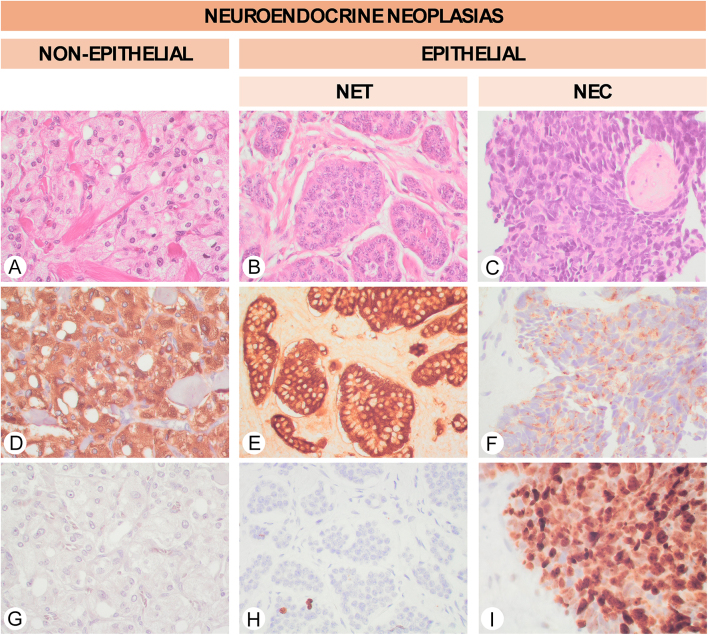
Spectrum of neuroendocrine neoplasia. (A) Medullary paraganglioma (pheochromocytoma) showing a zellballen pattern composed of large cells with pale and granular eosinophilic staining (H&E, 40×). Paraganglioma is characterized by the expression of neuroendocrine markers, such as chromogranin (D, chromogranin-A, 40×), and the absence of expression of keratins (G, CKAE1/AE3, 40×). (B) Well-differentiated neuroendocrine tumor (NET) composed of nests surrounded by a collagenous stroma (H&E, 40×). (C) Small cell carcinoma is a subtype of neuroendocrine carcinoma composed of cells with a high nucleus-to-cytoplasm ratio together with frequent mitosis and apoptotic bodies (H&E, 40×). The NET has a strong cytoplasmic expression of chromogranin (E, chromogranin, 40×) and a low proliferation index (H, Ki67, 40×). In contrast, small cell carcinomas show a granular punctuated expression of neuroendocrine markers (F, chromogranin, 40×) and a high proliferation index (I, Ki67, 40×).

The classification of NENs has greatly evolved in the past decade, bringing together advances in the molecular biology of these tumors with clinical implications. NENs comprise a varied group of neoplasms characterized by the presence of neurosecretory granules (a characteristic histology) and the expression of neuroendocrine markers, including the well-recognized neuroendocrine markers synaptophysin and chromogranin, which stain granules present in the cytoplasm. In addition, the transcription factor of neuroendocrine differentiation, insulinoma-associated protein 1 (INSM1), is increasingly used in the clinic to assess neuroendocrine differentiation (Rosenbaum *et al.* 2015, Zhang *et al.* 2021). NENs can be divided into two main groups (Fig. 3): epithelial NENs and non-epithelial NENs, being differentiated by the expression (or not) of epithelial markers, such as keratins, whose positive expression is commonly used to classify a NEN in the epithelial subgroup. Non-epithelial NENs are composed of paragangliomas (both extra adrenals and adrenals, also called pheochromocytomas) and will not be discussed in this review (Rindi *et al.* 2022).

Epithelial NENs can be further divided into two different groups: NETs and neuroendocrine carcinomas (NECs) (Fig. 3). Although they were considered closely related neoplasms, an issue that has created a great level of confusion at the level of terminology, they are currently considered biologically independent entities (Rindi *et al.* 2018). What is more important to consider is that both groups have differences in their histological appearance, biological behavior, prognosis, and treatment. To solve terminology-related problems, a common classification for NENs was proposed in 2018 (Rindi *et al.* 2018). NECs are currently pathologically defined by a characteristic morphology (further reviewed in Rindi *et al.* (2018)), highlighted by a mostly solid sheet pattern of growth, with a high proliferation rate, frequent necrosis, and numerous apoptotic bodies (Fig. 3C). Molecularly, NECs frequently have alterations in TP53 and/or RB1 genes (Uccella *et al.* 2021). In the context of other solid tumors, neuroendocrine differentiation has been identified. For prostate cancer, this histological differentiation has been more readily studied, resulting in its particular classification due to its clinical impact, which has resulted in new treatment approaches. Indeed, prostate NECs represent an aggressive histological variant typically seen in advanced stages of prostate cancer, often as a response to treatment resistance, known as treatment-emergent neuroendocrine prostate carcinoma (t-NEPC). Thus, dedifferentiation/selective pressures mediated by treatments can give rise to t-NEPCs. While this can occur in other organs, it appears to be more common and better studied in the prostate and, therefore, has attracted more clinical attention (De Kouchkovsky *et al.* 2024).

In contrast, NETs are well-differentiated neoplasms with an organoid pattern of growth. They are currently further divided into three grades using parameters such as the amount of mitosis per 2 mm^2^, the proliferation index assessed by Ki67 immunohistochemistry, and the presence of necrosis in some organs. Notably, the potential to metastasize or invade adjacent tissues depends on the tumor site and grade (Rindi *et al.* 2018). This association between site and behavior explains why, although a common framework for classification was intended, grading is still performed according to the site of origin. For example, the cutoff points used for NETs in the digestive system are grade 1 (<2 mitosis/2 mm^2^ and Ki67 proliferation index <3%), grade 2 (2–20 mitosis/2 mm^2^ or Ki67 proliferation index 3–20%), and grade 3 (>20 mitosis/2 mm^2^ or Ki67 proliferation index >20%) (Klimstra *et al.* 2019). In contrast, lung NETs are still named as typical or atypical carcinoids based on the number of mitosis (with a cutoff of <2 mitosis/2 mm^2^) and the presence of necrosis (Travis *et al.* 2022). These changes in terminology in the past decades make it difficult to assess the real prevalence and prognosis of NETs in historical databases (Sonbol *et al.* 2022) and should be taken into consideration when reading publications addressing the old classifications. For example, some cell lines generally considered as ‘carcinoids’ (such as P-STS and QGP-1) have been shown to have TP53 mutations, more consistent with current NECs (Hofving *et al.* 2018).

Definitely, pathology-based analyses are mandatory for the proper diagnosis of biopsies or cytology, together with a Ki67 immunohistochemical quantification (Pavel *et al.* 2020). However, because of the widespread expression of somatostatin receptors in NETs, specifically SST_2_ and SST_5_, NETs can also be identified quite well using nuclear medicine imaging techniques, such as somatostatin receptor scintigraphy (Kwekkeboom *et al.* 2009). Altogether, the advances achieved over the past decades, at the level of diagnosis and the classification of NETs, have significantly improved our understanding of these tumors. While diverse, as previously mentioned, the most clinically relevant types of NENs include pancreatic NENs (PanNENs), due to their prevalence and varied clinical presentations, ranging from indolent to aggressive behavior. NETs originating from the gastrointestinal tract and lungs are frequently characterized by the secretion of serotonin, causing symptoms such as flushing and diarrhea.

Overall, NETs exhibit diverse clinical manifestations and require a multidisciplinary approach for optimal management, considering their variable aggressiveness and hormone secretion patterns. With this in mind, it would not be surprising if some of the definitions and properties used to identify, characterize, and validate the existence of CSCs in other solid tumors may not be applicable to NENs. The reader will find that many of the studies detailed below are inconclusive, and similar studies on the same tumor type are often contradictory and reach opposing conclusions. This may indeed be due to our attempts to fit NEN CSCs into our rigid predefined CSC definitions. The advent of more advanced omic-based approaches, such as scRNAseq and spatial transcriptomics, may provide more sophisticated ways to definitely prove the existence of CSCs in NENs, without the need for (or in combination with) more traditional CSC validation studies (e.g., *in vivo* tumorigenesis assays). Until then, we can only learn from what we know to date about CSCs in NENs.

Later, we discuss the current state of CSCs in NETs of the gastrointestinal tract, PanNENs, and prostate NECs, the latter due to their particularity, biological singularity, and clinical relevance, with the hope of further propelling research in the NEN CSC field. Indeed, there are a number of papers investigating CSCs in pituitary adenomas. However, we have not discussed this tumor type in this review as there exist significant differences between pituitary adenomas and NETs. In fact, this is a point of debate in the field, which has been nicely reviewed by Ho and coworkers in a 2023 *Nature Reviews Endocrinology* perspective article (Ho *et al.* 2023).

## CSCs in gastroenteropancreatic NETs

The gastrointestinal tract represents the primary location for NETs, followed by the pulmonary tract (Anaizi *et al.* 2015, Riihimaki *et al.* 2016, Oronsky *et al.* 2017, Bloemen *et al.* 2022). As NETs of the gastrointestinal tract vary and encompass a wide range of tissues (gastroenteropancreatic tract, small intestine, pancreatic gland, etc.), these tumors are classified together as gastroenteropancreatic NETs (GEP-NETs), whose frequencies vary based on country, region, and patient datasets used (Park *et al.* 2023). Data regarding the annual incidence of GEP-NETs highlight a steady increase over the past two decades (Xu *et al.* 2021), with a clear correlation between aggressiveness and poor prognostic outcomes, which may be linked to a CSC phenotype, as originally suggested in 2012 by Grande and coworkers in a very extensive review on the existence (or not) of CSCs in GEP-NETs (Grande *et al.* 2012). Thus, herein we will update what advances have been made regarding CSCs in GEP-NETs over the past 12 years; however, PanNENs are separately discussed from other GEP-NETs.

Before 2011, the presence of CSCs in GEP-NETs had not been experimentally demonstrated. Rather, the activation or expression of embryogenic and/or fetal developmental pathways (or related proteins), such as Hedgehog (Shida *et al.* 2006, Fendrich *et al.* 2007), Wnt/β-catenin (Su *et al.* 2006), and TGF-β (Chaudhry *et al.* 1993), had been observed in some GEP-NETs, indirectly suggesting that stem-like cells or stem-related pathways may be playing a role in NET development, chemoresistance, or survival (Grande *et al.* 2012). In late 2011, however, Gaur and coworkers identified ALDH-positive cells by immunohistochemistry in 14 NETs, ranging from 0.5 to 20.1% (Gaur *et al.* 2011). Using the cell line CNDT2.5 (Van Buren *et al.* 2007), the authors were also able to sort ALDH+ cells and show that the positive population had greater sphere-forming capacity and *in vivo* tumorigenic potential than the ALDH-negative population. Importantly, since Src signaling was increased in ALDH+ cells, the authors experimentally demonstrated that Src represented a druggable target. Specifically, targeting Src with the known inhibitor PP2 *in vitro* or with siSrc DOPC-conjugated liposomes in mice xenografted with CNDT2.5 cells reduced ALDH levels and impeded tumor growth, with 20% of the tumors regressing with treatment (Gaur *et al.* 2011). Therefore, this was the first study to experimentally show the existence of CSCs in midgut NETs using CSC markers, *in vitro* functional assays, and *in vivo* xenograft models. The CSC marker CD133 has also been explored in GEP-NETs. Using a panel of 90 digestive tract NENs with their matched non-neoplastic mucosa, Mi-Jan and coworkers showed that CD133 was expressed in 26.1% of poorly differentiated NECs and 30.3% of well-differentiated NETs, although no correlation with tumor grade, site, expression of neuroendocrine markers, or patient survival was observed (Mia-Jan *et al.* 2013). This is in contrast to what has been observed for other tumors, such as pancreatic ductal adenocarcinoma (PDAC), where CD133 has a prognostic value (Li *et al.* 2015, Poruk *et al.* 2017) and correlates with a pro-tumorigenic gene expression profile (Skoda *et al.* 2016).

As detailed by Grande and coworkers, Notch signaling in GEP-NETs has been a focus of research for some time (Grande *et al.* 2012), and while Notch has been linked with CSC ‘stemness’ in diverse tumors, such as colorectal cancer (Brisset *et al.* 2023), PDAC (Bao *et al.* 2011, Bailey *et al.* 2014), and breast cancer (Jiang *et al.* 2020), its role in GEP-NETs was believed to be antitumoral rather than pro-tumoral. This is due to the fact that activation of Notch-1 signaling is correlated with tumor suppressor properties in different GEP-NET cell lines and in medullar thyroid carcinoma and small cell lung cancer (Kunnimalaiyaan & Chen 2007, Chung & Xu 2023). Thus, the conclusion was that Notch-1 inhibition would not be beneficial for GEP-NETs (Chung & Xu 2023). However, a 2013 study by Wang and coworkers, using 120 well-differentiated NETs, including tumors originating in the pancreas, ileum, and rectum, showed Notch-1 heterogeneity across tumors, with Notch-1 immunohistochemical staining being uniformly expressed in rectal NETs (100%) and a subset of PanNETs (34%) and negative in ileal NETs. While the authors did not link Notch-1 expression with CSCs, the study does highlight that this known CSC-signaling pathway is expressed in a subset of GEP-NETs (e.g., rectal NETs), and Notch-1 inhibitors may have clinical utility is certain GEP-NETs (Wang *et al.* 2013). Similar to Notch, Wnt/β-catenin signaling is also strongly linked to a CSC phenotype (Kahn 2018, Martin-Orozco *et al.* 2019), and the role of this signaling cascade in NETs has been an area of interest for decades, again with inconclusive results. In the past decade, however, studies have emerged showing that Wnt/β-catenin signaling is indeed relevant in GEP-NETs, and targeting this signaling cascade can have negative effects on NETs (Kim *et al.* 2013, 2015, Wei *et al.* 2018). For example, using the human small intestinal NET cell line GOT1, Jin and coworkers showed that WNT974, an inhibitor of Porcupine (PORCN), a membrane-bound O-acyl transferase required for the palmitoylation of Wnt proteins and essential in diverse Wnt pathways (Covey *et al.* 2012), had antitumor effects in NET cell lines via suppression of Wnt and associated signaling pathways. Moreover, the β-catenin inhibitor PRI-724 also exhibited antitumor properties in NET cell lines (Jin *et al.* 2020). Interestingly, Wnt signaling regulates MYC expression (He *et al.* 1998), and Griger and coworkers have recently published an elegant genome sequencing study to characterize the genomic landscapes of human GEP-NECs and histologic variants, showing gains of MYC family members in a large part of the analyzed cases (Griger *et al.* 2023). The link between MYC and CSCs is well established (Elbadawy *et al.* 2019), and therefore, Wnt/β-catenin activation in GEP-NECs may also facilitate a CSC phenotype via MYC activation.

In summary, while more specific CSC-based assays are still needed to unequivocally prove the existence of CSCs in GEP-NETs, indirect evidence supports that CSCs and CSC-related pathways play a role in GEP-NETs. It is important to note that while the majority of the aforementioned studies do not specifically mention CSCs, they do touch upon pathways that are linked to the CSC phenotype and, therefore, indirectly support the CSC model in GEP-NETs. Similarly, an extensive review by Aristizabal Prada and Auernhammer detailed the molecular targeted therapies for GEP-NETs, highlighting targetable CSC-associated signaling pathways, including ‘PI3K, Akt, mTORC1/mTORC2, GSK3, c-Met, Ras–Raf–MEK–ERK, embryogenic pathways (Hedgehog, Notch, Wnt/β-catenin, TGF-beta signaling, and SMAD proteins), tumor suppressors and cell cycle regulators (p53, cyclin-dependent kinases 4 and 6 (CDK4/6), CDK inhibitor p27, and retinoblastoma protein), heat shock protein HSP90, aurora kinase, Src kinase family, focal adhesion kinase, and epigenetic modulation by histone deacetylase inhibitors’ (Aristizabal Prada & Auernhammer 2018).

## CSCs in PanNENs

The existence of CSCs in PanNENs has been under debate for more than a decade (Grande *et al.* 2012), and probably due to insufficient research, CSCs have not been clearly identified and/or properly isolated from PanNENs. Nevertheless, there is increasing indirect evidence of either their existence in these tumors or an activation of pathways related to stem cells and pancreatic development, highlighted by the number of studies showing the presence of stem cell markers in PanNEN cells. Moreover, the development of new models, such as organoids, should allow us to better understand the cell composition and necessities of PanNENs, including those related to CSCs (Kawasaki *et al.* 2020).

Classically, PanNENs have been characterized by alterations in signaling pathways strongly associated with cell differentiation, development, and stem cells, both normal and tumoral, as is the case for Notch, Sonic Hedgehog, or Wnt/β-catenin. However, these pathways were linked to tumorigenesis, tumor aggressiveness, and progression independently of a direct CSC link (Frost *et al.* 2018, Chung & Xu 2023). There are some exceptions, as is the case of INSM1, a transcriptional regulator necessary for endocrine differentiation of the pancreas (Gierl *et al.* 2006, Liang *et al.* 2021), which has been previously related to NETs (Mahalakshmi *et al.* 2020). It was shown that this factor may be crucial for determining the fate of PanNETs, since it may define whether a tumor develops into an insulinoma or a non-functioning PanNET (NF-PanNET), which is of clinical importance due to the greater aggressiveness/metastatic capacity of NF-PanNETs. Specifically, Kobayashi and coworkers used the RT2 B6 mouse model for insulinoma, which when bred into a hybrid AB6F1 genetic background, produced NF-PanNETs that the authors linked to repression of Insm1 (Kobayashi *et al.* 2019). This link was based on the higher metastasis and lower insulin expression observed when Insm1 was altered and the hypermethylation of its promoter in NF tumors. Similarly, they showed that when knocked down in cell lines, stem cell markers (ALDH and CD44) and stem-like behavior were promoted (Kobayashi *et al.* 2019), indicating that Insm1 may be an important regulator of stemness and PanNET identity. Importantly, INSM1 has been related to p53, MEN1, and Notch and has also been connected to modulation of the classic CSC markers CD133 and FOXA2 in cell lines (Capodanno *et al.* 2021).

In this context, over the past years, more specific CSC markers have been detected in PanNENs, moving the field closer to definitively demonstrating their existence. In the same aforementioned study by Gaur and coworkers, ALDH was also used to isolate ALDH-positive cells (0.2–5.9% of total cells) with the ALDEFLUOR assay from fresh PanNEN samples, in addition to other GEP-NETs. ALDH-positive PanNEN cells generated more tumors with faster kinetics than the ALDH-negative population *in vivo*, satisfying the main CSC requirement (i.e., tumor-initiating capacity) and showing for the first time the possible existence of a stem cell component in PanNENs (Gaur *et al.* 2011). However, the authors mention that the putative ALDH-positive CSC population did not overlap with CD44- or CD133-positive cells, two of the most commonly used markers for CSC detection. This was also the case for the study led by Katsuta and coworkers, where a cell population with high ALDH expression was linked with CSC features, specifically sphere-forming capacity, tumorigenicity, and CD73 expression, although neither CD44 nor CD133 was overexpressed in this population (Katsuta *et al.* 2016). In line with this, Mia-Jan and collaborators found CD133 immunoreactivity in PanNENs, while normal islets were negative for CD133; however, only two samples were included in the study and only one was positive, making it difficult to draw conclusions from these results (Mia-Jan *et al.* 2013). More recent studies, however, have shown that CD133 and CD44 were present in a cohort of 71 PanNENs and associated with worse prognosis (Sun *et al.* 2020) and that CD133 was linked to a more aggressive phenotype in two independent cohorts of 178 and 56 PanNENs (Sakai *et al.* 2017), but no specific or further association with CSCs was made. Another well-studied stem cell marker is DCLK1, which was first described in intestinal and PDAC tumors (Sureban *et al.* 2011, Nakanishi *et al.* 2013). Although Fan and coworkers found that this gene was not expressed in any of the 22 PanNETs included in their study (Fan *et al.* 2020), an independent study from Ikezono and collaborators showed that DCLK1 was highly and diffusely expressed in all PanNEN samples analyzed, and its overexpression in cell lines led to EMT and a more aggressive phenotype (Ikezono *et al.* 2017). PanNETs (and NENs in general) are characterized by a remarkably high heterogeneity (Pedraza-Arevalo *et al.* 2018), which, added to the very limited patient cohorts (22 and 15, respectively), could be the reason why these two studies found completely different results. Krampitz and collaborators identified a cell population with high expression of CD90 (a marker of hematopoietic stem cells) in patient-derived primary tumors, which overlapped with enhanced expression of ALDHA1, upregulation of stem cell genes, and increased xenograft tumor development, thus highlighting these CD90-positive cells as putative tumor-initiating cells and/or potential CSCs in low-grade PanNETs (Krampitz *et al.* 2016). In 2015, Salaria and collaborators showed that both small intestine tumors and PanNENs express CD24, a stem cell marker present in normal and tumoral (e.g., PDAC) stem cells (Salaria *et al.* 2015). Islets did not express this marker, and while 5% of included PanNENs exhibited strong subnuclear CD24 staining, the majority of the tumor cells stained positive, thus reducing the possibility that these CD24-positive cells represented a scarce population of PanNEN CSCs. More recently, Guo and coworkers have shown that PKD1 is highly expressed in CD44-positive putative PanNET CSCs (Guo *et al.* 2022) and that signaling derived from this gene may regulate a specific subpopulation of CSCs, characterized by a partial EMT phenotype.

One of the main targeted treatments available for PanNETs is sunitinib, a multitargeted tyrosine-kinase inhibitor that acts on different molecules simultaneously and has shown clear antitumor effects (Papaetis & Syrigos 2009). One of its targets is c-KIT (stem cell factor receptor or CD117), which is differentially expressed on stem and tumor cells (Hassan 2009). Its expression has been associated with more aggressiveness in NENs, including PanNENs, specifically shorter disease-free survival (Knosel *et al.* 2012). Together with KRT19 (a structural protein present in exocrine and developing but not mature endocrine pancreas), they have been suggested as poor prognosis biomarkers (Han *et al.* 2013), although this was not supported in different types of PanNENs (Cherenfant *et al.* 2014). In 2020, Lee and collaborators reviewed the effect of c-MET inhibitors in different types of PanNENs (Lee *et al.* 2020). They showed that c-MET overstimulation increased xenografted PanNETs in mice, and none of those tumors survived in the absence of c-MET expression. *In vitro* and *in vivo* assays also demonstrated that the aggressiveness and metastasis of PanNENs may be dependent on c-MET (Lee *et al.* 2020). Although these studies do not mention CSCs, c-MET is a CSC surface marker (Miekus 2017); thus, the effect of its inhibition indirectly associates c-MET and CSCs. The latter is further supported by the positive effects of the c-MET inhibitor, cabozantinib, showing over 20 months of progression-free survival in a clinical trial (Lee *et al.* 2020).

A different approach was used by Capodanno and coworkers in 2018, where they tried to characterize and target CSCs in cells derived from an insulinoma, an insulin-secreting tumor and one of the most common PanNET types. They showed that *in vitro* spheres exhibited higher expression of stem cell markers, such as CD34, CD133, OCT4, SOX2, and SOX9, and the Notch-related genes NOTCH2, HES1, and HEY1 compared to parental adherent cells. These spheres also showed greater resistance to 5-FU treatment and more invasive and tumorigenic capacity (Capodanno *et al.* 2018). Similarly, Buishand and coworkers used INS and BON-1 cells to search for stem cell markers and found that CD90-positive cells, but not CD166- or GD2-positive cells, exhibited greater tumor initiation capacity in xenografted mice and anti-CD90 treatment decreased cell viability and metastatic potential in xenografted zebrafish (Buishand *et al.* 2016). In 2021, a case report published by Venugopal and coworkers analyzed the stem cell phenotype of a well-differentiated G1 PanNET (Venugopal *et al.* 2021). This study showed a correlation between mRNA and protein expression of several stem cell markers, including CD24, CD44, and CD49, with the first two increased in tumors. However, as in other studies, the possibility of the existence of a CSC population was not considered. Along these lines, a 2015 study by Sadanandam and collaborators used transcriptomic analyses to distinguish between metastasis-like and other PanNENs, showing that the metastasis-like tumors exhibited greater stemness, although, again, they did not link these properties to the existence of a CSC population (Sadanandam *et al.* 2015). In summary, there are several available studies about CSCs in PanNETs although most of them do not specifically consider these cells as a main focus. Many can be classified into four different approaches: i) focused on stem cell markers and their function but not considering stem cell populations (e.g., INSM1); ii) trying to demonstrate the existence of a stem cell population in these tumors using classical markers (e.g., ALDH, DCLK1, CD90, and CD24); iii) exploring the effect of PanNEN treatments on stem cell features (e.g., c-KIT and c-MET inhibitors); and iv) depicting phenotype of stem cells in different models. Nevertheless, the existence of CSCs and their putative role in PanNENs is still widely unclear, and thus, further studies are still needed to definitively claim the existence of CSCs in these tumors.

## Prostate CSCs in neuroendocrine differentiation

Neuroendocrine cells constitute a differentiated and less represented epithelial cell compartment in normal prostate (<1%) (Butler & Huang 2021). Nevertheless, most cases of adenocarcinoma from the prostate also include a component of neuroendocrine proliferation with no expression of androgen receptors and prostate-specific antigen. This subpopulation of cells has progressively acquired more interest in recent years. Indeed, an increasing number of cases with a neuroendocrine component have been defined in patients with prostate cancer in different clinical and treatment scenarios, with an enrichment in advanced treated disease, from less than 2% at the initial diagnosis to 10–30% in the castration-resistant setting. This progression, which was variable between patients, has been suggested to originate from clonal selection heterogeneity or stemness in prostate cancer cells due to prolonged androgen deprivation therapy. As an extreme situation, the small cell NEC is the most aggressive variant in prostate cancer with a poor prognosis in patients at *de novo* diagnosis, with a median overall survival of less than 2 years (Beltran *et al.* 2014).

Neuroendocrine cells in the prostate are characterized by the expression of p63, 34βE12, CK5/6, CK14, and specific markers different from secretory or basal cells, such as chromogranin A, synaptophysin, calcitonin, CD56, neuron-specific enolase, FOXA2, and CXCR2, but lack ARs and PSA expression (Butler & Huang 2021). However, NETs of the prostate are exceptionally rare.

CSCs have been suggested to be involved, among other factors, in relapse and resistance to therapy in prostate cancer in relation to their lineage plasticity, heterogeneity (epithelial vs mesenchymal), and ability for transdifferentiation to a neuroendocrine subtype (Han *et al.* 2022). Different CSC markers are expressed in prostate CSCs (such as CD44, CD133, CD49f, α2/β1, EpCAM, CD117, SCA-1, CD54, ABCG2, CXCR4, E-cadherin, SOX2, Nanog, OCT4, SUZ12, BMI-1, β-catenin, P63, ALDH1A1, EZH2, and TDGF1), but some of them are particularly related to neuroendocrine differentiation (Verma *et al.* 2023). SOX2 expression has shown to be inversely regulated by the lack of androgens in the context of prostate cancer therapy. This pluripotency control factor favors tumor transdifferentiation via lncRNAs, such as H19, involved in neuroendocrine-related gene expression by epigenetic regulation. In addition, SOX2 can cooperate with another transcription factor, N-MYC, for CSC regulation and promotion of this aggressive prostate cancer subtype associated with *TP53* and *RB* loss (Dardenne *et al.* 2016, Verma *et al.* 2023). In this sense, N-MYC promotes polycomb repressive complex 2 (PRC2) signaling and enhances EZH2 and AURKA expression, related to cell migration and invasiveness (Dardenne *et al.* 2016). Furthermore, CD44 expression has been associated with neuroendocrine features in prostate cancer cell lines (Palapattu *et al.* 2009, Singh *et al.* 2021, Zhou *et al.* 2023*a*). However, it seems that other neuroendocrine phenotype drivers apart from CSC markers, such as BRN2/4, MUC1-C, SIAH2, or ONECUT2, may have a role in this process of differentiation (Guo *et al.* 2019). Recently, Cheng and coworkers have identified multipotent stem-like cells in primary tumors of the prostate, not previously treated, using single-cell RNA sequencing. They provided a novel mechanism of disease progression and aggressiveness different from adaptation to treatment pressures (Cheng *et al.* 2022).

Interestingly, some signaling pathways have been suggested to maintain prostate CSC homeostasis, such as Hedgehog, Wnt, Notch, Hippo, PI3K/AKT, AP1, NF-κB, or JAK-STAT upregulation and AR downregulation, and therefore represent selective targets for therapeutic strategy development, but are currently at early stages (Verma *et al.* 2023, Zhou *et al.* 2023*a*). In addition, the CSC tumor niche/microenvironment represents another potential target to regulate CSCs and to overcome treatment resistance to current therapies (Zhou *et al.* 2023*a*). However, more research is needed on CSC characterization to understand their involvement in the divergent development of neuroendocrine differentiation or castration-resistant progression and whether there is a relationship between both evolution patterns, in order to provide better information on potential treatment targets/strategies (Germann *et al.* 2012, Verma *et al.* 2023).

## Clinical relevance of CSCs in NENs and conclusions

The current treatment for NENs include somatostatin analogs, VEGFR- and mTOR-targeted agents, peptide receptor radionuclide therapy, and chemotherapy based on alkylating agent- or platinum-based schedules (Alabi *et al.* 2022, Li *et al.* 2022, Modica *et al.* 2022). These treatments have shown efficacy in different types of NENs, such as GEP-NENs and bronchopulmonary NENs (Hijioka *et al.* 2021, Garcia-Carbonero *et al.* 2023). The choice of treatment depends on factors such as tumor origin, grade of differentiation, stage, radiologic or metabolic images, and other patient characteristics. However, resistance to chemotherapeutic agents remains a major limitation in the clinical application of these treatments and further potentially druggable targets are urgently needed. Thus, the goal is to achieve individualized personalized treatment strategies for NETs, focusing on optimal benefit populations and treatment sequence strategies (Kiesewetter & Raderer 2020).

Applying what we know and have learned from CSC-targeted therapies to NETs could be beneficial. Recent reviews have outlined potential strategies for eradicating CSCs in other tumor entities (Chen *et al.* 2013, Ning *et al.* 2013, Saygin *et al.* 2019), which encompass i) targeted therapy aimed directly at eliminating CSCs, ii) inducing an active cell cycle to render quiescent CSCs susceptible to treatment, or iii) disrupting the CSC niche and/or TME. The main issue in applying these therapies to NETs is our still immature understanding of NET CSCs in general and, even more so, our almost incomplete knowledge regarding the NET CSC niche, the TME, and the communication between NET CSCs and the cells present within the TME (Cuny *et al.* 2022). Similarly, we are far from understanding the metabolic requirements of NET CSCs. For example, in PDAC, we have discovered that PDAC CSCs depend on mitochondrial oxidative phosphorylation (OXPHOS) to meet their energy needs (Sancho *et al.* 2015, Valle *et al.* 2020), and targeting CSC OXPHOS in PDAC, as well as colorectal and osteosarcoma, is a therapeutically viable approach (Alcala *et al.* 2024). However, whether a metabolic inhibitor would be beneficial for treating NETs is still unknown. Finally, similar to CSCs of other tumors (Cioffi *et al.* 2015, Alvarado *et al.* 2017), it is reasonable to assume that NET CSCs may also promote immunoevasion. Thus, as immunotherapy is being tested in some NETs, it may be advantageous to study the immune checkpoint and immune evasion marker profile of NET CSCs. Advances on some of these fronts are being made in pituitary NETs, but not necessarily at the CSC level (Tapoi *et al.* 2023, Zhou *et al.* 2023*b*, Yan *et al.* 2024). Similarly, new technologies, such as spatial transcriptomics and single-cell RNAseq analyses of NETs, will also almost certainly advance our understanding of CSCs in NETs and the interrelation between cells that express CSC markers with other signaling pathways or biomarkers.

CSCs offer a promising research avenue to better understand tumor biology, evolution, chemoresistance, and metastasis and offer a target for developing new therapies for treating patients with NETs. The efficacy of emerging anti-NET CSC therapies, however, hangs on our ability to expand our knowledge of NET CSCs, their metabolism, immunoevasive properties, niche, and the CSC-TME crosstalk and our capacity to identify those individuals most likely to respond favorably to future anti-NET CSC therapies, perhaps with NET CSC-specific biomarkers that can be incorporated into routine pathology-based diagnostic systems for NETs. Table 1 summarizes the CSC markers discussed in the review, the CSC phenotype studied, and the tumor entity. Indeed, we have made advances in better diagnosing, classifying, and naming NETs, but we need to continue expanding our understanding of the role of CSCs in NETs. One of the main obstacles to overcome to achieve this goal is our need to fit NET CSCs into our traditional CSC models with our predetermined list of CSC markers and functional assays. NETs have proven to have unique clinical and biological characteristics that differentiate them from other tumor types (Smith *et al.* 2023), arising from classical endocrine organs and dispersed neuroendocrine cells. Thus, while applying what we know about CSCs from other tumors to NETs may be beneficial, it may at the same time be restrictive and counterintuitive based on the difference that exists between NETs and other tumor entities. Indeed, this philosophy may reconcile variances observed in a number of published NET CSC studies, such as the lack of colocalization between ALDH1 and traditional CSC markers, such as CD133 and CD44, in the study by Gaur and coworkers (Gaur *et al.* 2011). As healthcare professionals and researchers devoted to managing or studying NETs, it is imperative that we allocate additional time and resources to further study CSCs in NETs (and their TME) as they i) may unveil interesting new knowledge about CSCs and ii) hold potential as a future therapeutic target for patients with advanced NETs.

**Table 1:** CSC markers, phenotype, and associated NETs.

NET Type	Marker	Phenotype	Reference
Intestinal	ALDH	Spheres formation, tumorigenicity	Gaur *et al.* (2011)
Intestinal	CD133	-	Mia-Jan *et al.* (2013)
Intestinal	Notch1	-	Wang *et al.* (2013)
Intestinal	PORCN	Wnt activation	Jin *et al.* (2020)
Intestinal	MYC	Mutations	Griger *et al.* (2023)
Pancreatic	INSM1	NET fate determination, stem-like behavior inhibition	Kobayashi *et al.* (2019)
Pancreatic	INSM1	Modulation of CSC markers	Capodanno *et al.* (2021)
Pancreatic	ALDH	Spheres formation, tumorigenicity	Gaur *et al.* (2011)
Pancreatic	ALDH	Spheres formation, tumorigenicity	Katsuta *et al.* (2016)
Pancreatic	CD133	-	Mia-Jan *et al.* (2013)
Pancreatic	CD133/CD44	Worse prognosis	Sun *et al.* (2020)
Pancreatic	CD133	More aggressive	Sakai *et al*. (2017)
Pancreatic	DCLK1	EMT, higher aggressiveness	Ikezono *et al.* (2017)
Pancreatic	CD90	Stem cells genes, tumorigenicity	Krampitz *et al.* (2016)
Pancreatic	CD24	-	Salaria *et al.* (2015)
Pancreatic	PKD1	EMT	Guo *et al.* (2022)
Pancreatic	c-KIT	Shorter survival	Knosel *et al.* (2012)
Pancreatic	c-KIT/KRT19 (CK19)	Poor prognosis	Han *et al.* (2013)
Pancreatic	c-MET	Tumorigenicity, shorter PFS	Lee *et al.* (2020)
Pancreatic	-	Spheres formation, tumorigenicity, treatment resistance	Capodanno *et al.* (2018)
Pancreatic	CD90	Tumorigenicity, metastatic potential	Buishand *et al.* (2016)
Pancreatic	CD24/CD44/CD49	-	Venugopal *et al.* (2021)
Pancreatic	-	Metastatic-like	Sadanandam *et al.* (2015)
Prostate	SOX2	Androgens presence, transdifferentiation	Verma *et al.* (2023)
Prostate	SOX2/N-MYC	Cell migration, aggressiveness	Dardenne *et al.* (2016)
Prostate	CD44	Neuroendocrine features	Singh *et al.* (2021)
			Palapattu *et al.* (2009)
			Zhou *et al*. (2023)
Prostate	ONECUT2	Neuroendocrine driver	Guo *et al.* (2019)

## Declaration of interest

The authors declare that there is no conflict of interest that could be perceived as prejudicing the impartiality of this work.

## Funding

This work did not receive any specific grant from any funding agency in the public, commercial or not-for-profit sector.
